# LED-Based Photometric Systems for Decentralized Chemical Analysis: Applications, Design Considerations, and Practical Limitations

**DOI:** 10.3390/s26196109

**Published:** 2026-09-26

**Authors:** Elisa Jekel Könnel, Roland Ulber, Lena Geuer

**Affiliations:** Department of Mechanical and Process Engineering, Division of Bioprocess Engineering, University of Kaiserslautern-Landau, 67663 Kaiserslautern, Germany; elisa.koennel@mv.rptu.de (E.J.K.); roland.ulber@mv.rptu.de (R.U.)

**Keywords:** LED-based photometer, portable analytical devices, low-cost spectroscopy, environmental monitoring, point-of-care testing (POCT), food quality analysis, process analytical technology (PAT)

## Abstract

Photometric methods are essential in analytical chemistry for environmental, biological, and industrial monitoring. Conventional spectrometers rely on deuterium or tungsten lamps, which are bulky, energy-intensive, and costly to maintain. Advances in light emitting diode (LED) technology have enabled compact, energy-efficient, and cost-effective alternatives for portable and decentralized chemical analysis. This review provides an application-oriented synthesis of recent developments in LED-based photometry, systematically comparing major analytical fields to identify common design strategies, recurring performance limitations, and emerging trends. Representative applications include environmental monitoring, point-of-care diagnostics, food quality control, industrial process analysis, and STEM education. Design-related aspects, including optical layouts, detector selection, wavelength matching, and calibration strategies, are discussed where they influence analytical performance and practical implementation. Recent innovations, such as multi-wavelength LEDs, 3D-printed housings, and microcontroller-based systems, have further expanded the accessibility and adaptability of LED-based photometers. Across the reviewed studies, progress has been driven primarily by translating established photometric methods into compact, application-specific platforms rather than by developing new analytical assays. Despite these advances, challenges remain regarding sensitivity, calibration robustness, validation, and methodological standardization. By identifying cross-cutting challenges and successful implementation strategies, this review provides guidance for the future development of robust, low-cost, and reliable LED-based photometric systems for decentralized chemical analysis.

## 1. Introduction

Analytical methods play a crucial role in addressing challenges across environmental monitoring, healthcare, industrial processes and education. Whether ensuring safe water and food, tracking pollutants, or optimizing production lines, reliable data is essential for informed decision-making. In educational settings, analytical tools also enhance learning by providing hands-on experience with measurement techniques and data interpretation. However, traditional analytical workflows often require manual sampling, transportation to centralized laboratories, and the use of complex, expensive instrumentation operated by trained personnel. These procedures can be time-consuming and costly, with a risk of compromising sample integrity during sampling, transportation, and storage. As global concerns such as resource scarcity grow, there is an increasing demand for portable, cost-effective tools that enable on-site, real-time analysis.

In this context, photometric techniques, particularly absorbance-based methods, offer a versatile solution for field-based analysis. By measuring light absorption, scattering, or emission, it enables quantitative analysis across diverse applications, from environmental science to clinical diagnostics. Historically, photometric systems were limited to laboratory settings due to their large size and high acquisition costs, limiting accessibility for small businesses, educational institutions, and resource-limited regions. Recent advances in light emitting diode (LED) technology have changed this. LED-based systems integrate the precision of photometric analysis with the compactness, affordability, portability, and durability of LEDs. By providing a practical and miniaturized solution, they enable advanced analytical capabilities in settings where traditional laboratory infrastructure is impractical, expanding access to critical data and its applications.

The concept of LED-based photometry was introduced in 1973 by Flaschka et al., who developed a simple cell module using a red LED and a phototransistor [1]. Since then, LED-based systems have evolved significantly, with the publication of numerous reports and studies exploring their general application in analytical chemistry [2,3,4,5,6,7,8,9,10]. Reviews by the group of Dasgupta about flow-through optical absorption detectors based on LEDs in 1993, 2003 and 2014 [11,12,13], by O’Toole and Diamond in 2007 [14] and by the group of Hauser in 2013 and 2015 [15,16] have summarized developments in this field. While these reviews primarily focused on detector concepts and instrumental configurations, the rapid expansion of LED-based photometric systems into diverse analytical applications over the past decade has not yet been comprehensively assessed.

The present review was conducted as a narrative, application-oriented review, comparing developments across the major fields of environmental monitoring, healthcare, food quality control, process analysis, and education to identify common design strategies, recurring technological challenges, and emerging trends relevant to decentralized photometric analysis. Design-related aspects are discussed where necessary to explain analytical performance, while current technological challenges, research gaps, and future directions are highlighted. The defined literature search was conducted for the application-oriented comparative analysis presented in Section 3 and was designed to provide a broad overview of LED-based photometric applications. Section 2 provides historical background, describes technological developments, and discusses general design principles. Consequently, the publications cited in Section 2 are not part of this defined dataset and were therefore not subject to the 2015–2025 publication-period criterion. For the application-based analysis, the literature search was conducted using the Web of Science, Scopus, and Google Scholar databases. The final search was performed in January 2026. The search covered publications published between 2015 and 2025 and used combinations of keywords including “LED photometer”, “LED-based photometer”, “portable photometer”, “LED colorimeter”, and “LED fluorimeter”, supplemented by application-specific terms such as “low-cost”, “environmental”, “health”, “health monitoring”, “point-of-care testing (POCT)”, “food”, “food monitoring”, “industrial monitoring”, “product quality”, “product quality monitoring”, and “education”. Journal articles and relevant conference publications published in English between 2015 and 2025 were considered. Titles and abstracts were screened for relevance, followed by full-text assessment where necessary. Publications were included when they described analytical systems employing LEDs as optical excitation or illumination sources. Additional relevant studies were identified through citation tracking. Publications focusing exclusively on chromatographic detectors were excluded. For each included study, information on the analytical application, optical configuration, detector technology, employed LEDs, and analytical performance was extracted and synthesized. Each publication was assigned to a single primary application field according to its main intended application and was counted only once. Classification was based on the application context described by the authors. Direct quantitative comparison across studies is limited by substantial differences in analytes, assay chemistries, sample matrices, optical configurations, and reporting metrics. Analytical performance is therefore reported using the metrics and units of the original studies rather than normalized to a common scale. Accordingly, the review focuses on comparative technological and application-related trends rather than statistical aggregation across heterogeneous analytical systems.

## 2. Device Design and Measurement Principles of LED-Based Analysis Systems

### 2.1. Measurement Principles of LED-Based Analysis Systems

Spectrometric measurements rely on the interaction of light with matter, typically quantified through absorbance or fluorescence. In absorption-based analysis, light intensity is measured before and after passing through the sample, and concentration is inferred according to the Beer–Lambert law [17]. Fluorescence detection instead monitors emitted light at longer wavelengths after excitation, usually in the ultraviolet (UV) or visible range. Conventional spectrometers use broadband tungsten or deuterium lamps combined with monochromators, which increases size and complexity. In contrast, LED-based systems employ fixed-wavelength, quasi-monochromatic emitters, eliminating the need for wavelength-selection optics and enabling compact, low-power instrumentation. A simplified comparison between classical monochromator-based spectrophotometers and LED-driven photometric setups is provided in Figure 1.

The first LED-based analytical device reported by Flaschka et al. demonstrated simple absorption measurements in a compact, battery-powered format, laying the groundwork for modern portable photometers [1]. Early applications primarily relied on batch measurements in cuvettes, as illustrated by Imasaka et al., who used an LED-based setup for phosphate determination via the molybdenum blue method [8]. Beyond batch measurements, LEDs have been widely integrated into flow-based systems such as flow-injection analysis (FIA) and multicommuted flow analysis (MCFA) [18,19,20,21]. These approaches use programmable valves and, in portable setups, compact solenoid micro-pumps to automate reagent handling, mixing [22], and cleaning steps, enabling high-throughput operation with minimal manual intervention [16,23,24]. Their reduced reagent consumption and waste generation also align well with environmentally oriented analytical practices [25,26,27]. While these characteristics are not exclusive advantages of LED-based detection, LEDs complement flow-based analytical systems particularly well and support their implementation in compact and portable formats.

LEDs are also widely used in fluorescence spectroscopy, where blue or UV LEDs typically serve as excitation sources due to the short wavelengths required for most fluorophores. High-intensity LEDs are advantageous because fluorescence sensitivity scales with excitation power. In addition to LEDs, laser diodes are increasingly employed as excitation sources and, with the exception of UV devices, are generally available at relatively low cost. Owing to their high optical power and narrow spectral bandwidth, laser diodes are particularly attractive for fluorescence excitation. However, depending on the laser design and operating conditions, they may exhibit higher intensity fluctuations than LEDs, which can negatively affect fluorescence signal stability unless appropriate stabilization or signal processing is applied [28,29]. Early demonstrations of the use of LEDs in fluorescence measurements include chlorophyll fluorescence measurement using a red LED as a light source [30] and a green-LED fluorimeter [31]. Today, LED-based fluorimeters are applied in diverse formats, including as on-column detectors in capillary electrophoresis, as reviewed extensively by Xiao et al. [32,33]. Advances in device fabrication have also contributed to the development of LED-based analytical systems. Beyond the selection of LEDs and detectors, practical performance depends on the mechanical and optical integration of these components. Reproducible positioning of the light source, sample cell, and detector is important for maintaining a consistent optical path, while light-tight housings or enclosed optical compartments reduce interference from ambient light. The increasing availability of consumer 3D printers enables the design of inexpensive, customizable housings that improve light shielding and reduce overall construction costs, particularly for educational or low-budget analytical tasks [34,35,36,37,38,39]. Such systems are often described as “homemade” or “DIY photometers,” reflecting both their simplicity and the growing interest in self-built scientific instrumentation. For portable systems, mechanical design must additionally consider device dimensions and weight, power supply, protection of optical and electronic components, and practical sample handling.

Today, LED-based photometers are also available as commercial instruments, offered by suppliers such as Macherey-Nagel, Hach-Lange, and Hanna Instruments. These devices are widely used for environmental monitoring, clinical diagnostics, and wastewater analysis [16]. While the widespread adoption of LED-based photometric instruments by established manufacturers highlights their practical usefulness when applied to well-defined analytical tasks, this does not imply universal applicability. When prototype LED photometers are compared with conventional commercial spectrophotometers, it should also be considered that these instruments are typically designed with different priorities. Their performance should therefore be evaluated not only in terms of analytical figures of merit but also with respect to application-specific requirements such as portability, cost, operational simplicity, and suitability for decentralized measurements. Agreement with a reference instrument for a specific analyte under defined test conditions should therefore not be interpreted as general analytical equivalence or as evidence of suitability for routine or field use. Accordingly, the performance of LED-based photometers strongly depends on careful system optimization, calibration strategies, and alignment with the specific analytical context. As a result, limitations frequently discussed in the literature reflect boundary conditions rather than fundamental inadequacy of the approach. In this review article, the term *LED-based photometric systems* refers to analytical systems that use LEDs as an optical excitation or illumination source for quantitative optical measurements, including absorption, fluorescence, and turbidity measurements, as well as related optical principles. Accordingly, this review covers various detector technologies and measurement configurations, provided that the LED constitutes the primary optical component of the measurement system. While LED-based devices have also been applied as optical detectors in chromatographic systems [40,41,42,43], these applications fall outside the scope of this review. Overall, the reviewed studies demonstrate that the versatility of LED-based photometry arises less from the measurement principle itself than from the ability to tailor optical and fluidic system designs to specific analytical tasks. This application-specific adaptability has been a major driver for the increasing use of LED-based photometers in decentralized analysis.

### 2.2. LEDs as Light Sources in Analysis Systems

After the first discovery of electroluminescence by the English inventor H. J. Round in 1907, it took several decades before LEDs became commercially viable [44]. In the 1960s, Texas Instruments released the first infrared (IR) LED with an emission wavelength of 890 nm. This was followed by the introduction of LEDs emitting in the visible spectrum: initially red, then orange, yellow, and green. The development of blue LEDs in the 1990s marked a technological breakthrough, completing the red–green–blue (RGB) tri-color set and enabling full-spectrum applications, including white light generation. This progression was driven by advances in semiconductor materials, as the wavelength of light emitted by an LED is determined by the bandgap energy of its semiconductor material: gallium arsenide (GaAs) for IR, gallium phosphide (GaP) for red/yellow/green, and indium gallium nitride (InGaN) for blue and green LEDs. Recent advances in AlGaN-based UV LEDs have enabled emission wavelengths down to 222 nm, with devices around 235 nm commercially available from major manufacturers such as Crystal IS [45,46]. While deep-UV LEDs are still relatively costly, their overall system cost remains lower than that of deuterium lamps including power supplies. Commercially available LEDs covering the ultraviolet (UV, 220–400 nm, visible (Vis, 400–700 nm) and near-infrared (NIR, 700–1550 nm) ranges are shown in Figure 2.

LEDs are often described as nearly monochromatic, typically emitting light within a narrow spectral range. Depending on the LED type and emission wavelength, typical full width at half maximum (FWHM) values are in the range of about 10 to 40 nm, with many visible LEDs having bandwidths of about 10 to 20 nm. In general, the spectral bandwidth tends to increase with increasing emission wavelength, although it also depends on the LED material system and device design. While this makes them “quasi-monochromatic” rather than strictly monochromatic [48], it is sufficient for many analytical tasks. However, the emission bandwidth of LEDs is generally wider than the spectral bandwidth typically selected for high-resolution, monochromator-based spectrophotometers, which often has an instrumental bandwidth of approximately 1 nm. In this respect, LED-based photometers are more similar to filter photometers or basic monochromator systems, in which measurements are likewise performed using broader spectral bands. Consequently, LED-based absorbance measurements are intrinsically polychromatic and may exhibit non-linear absorbance–concentration relationships across the measurement range. This behavior does not inherently compromise measurement accuracy when appropriate calibration models are applied, but it can reduce the effective dynamic range at higher absorbance values. The effects associated with the broad spectral emission of LEDs are partially compensated when an appropriate LED is selected such that its emission spectrum overlaps significantly with the absorption band of the analyte. As absorption spectra of molecular analytes in the UV–Vis range are generally broad, this overlap can be sufficient to ensure acceptable absorbance linearity and sensitivity. Depending on the application, multivariate or non-linear calibration models may further improve analytical performance by compensating for matrix effects and deviations from ideal Beer–Lambert behavior. Although these characteristics require appropriate wavelength selection and calibration strategies, LEDs have emerged as powerful alternatives to conventional light sources. Their compact size, energy efficiency, long operational lifespan, low heat generation, and low cost have enabled them to largely replace incandescent and discharge lamps in many systems [49,50,51], for example, for analytical photometric devices [2,52,53,54]. Furthermore, their emission wavelength is determined by the bandgap of the semiconductor material, allowing precise selection across a wide spectral range without the need for additional optical filters. Their modularity enables wavelength selection for specific analytical tasks, from UV absorption measurements to NIR probing of C–H, O–H, and N–H overtone vibrations. This spectral flexibility facilitates the development of portable, application-specific photometric devices. After a short thermal stabilization and with appropriate temperature compensation, LEDs provide stable optical output with low intensity noise. In absorbance measurements, however, an initial blank measurement (*I*_0_) is generally required to establish the reference intensity. This should be distinguished from continuous or dual-channel referencing, which is mainly required for applications demanding high long-term stability. In many portable single-beam LED photometers, reliable measurements can be achieved when the LED is operated under constant-current conditions and blank and sample are measured within a short time interval. Further aspects of signal acquisition and strategies for minimizing optical and electronic interferences are discussed in Section 2.3. When combined with appropriate electronics and data processing, such systems can also be adapted for real-time on-site analysis [13,55]. The practical advantages of LEDs, particularly their low power demand and durability, have been instrumental in driving the miniaturization of analytical instruments. Combined with recent trends in microelectronics, they support the development of compact, battery-operated devices for field and point-of-care applications. One inherent limitation of LEDs is their fixed emission wavelength, which restricts the ability to scan across a broad spectrum. To address this, several approaches have been developed. Some authors have solved this obstacle by using switchable multi-wavelength LEDs, which cover a wide range of the electromagnetic spectrum either simultaneously or individually and offer the advantage that no manual changing of LEDs is required [9,10,56,57]. A fiber optic bundle can be used to direct the light of several LEDs into the measurement cell [10]. To extend spectral coverage while maintaining simplicity and affordability, bi- or tri-color LEDs [58,59,60] or white LEDs in combination with a wavelength selective device [6,61,62,63] have been used. Beyond such approaches, more advanced architectures have been reported that focus on minimizing optical losses and improving signal fidelity. Notably, Kraiczek et al. [64] introduced an innovative optical architecture in which multiple individually addressable LEDs are efficiently combined at a common optical point using a reversed diffraction grating, thereby avoiding significant light losses associated with fiber coupling or beam splitting. In combination with analog log-ratio amplifiers or direct current digitization using devices such as DDC112, this approach enables direct absorbance measurement with reduced noise by integrating current digitalization within the detector electronics and eliminating the need for external signal-conditioning circuits and microcontroller-based logarithmic conversion [65,66]. Organic light emitting diodes (OLEDs) have also been investigated as alternative light sources in a small number of photometric studies [67,68,69,70,71]. Potential advantages of OLEDs include their thin design, mechanical flexibility and fast response time. However, they are generally more expensive than conventional inorganic LEDs and have a shorter service life. In photometric systems, for example, OLEDs have been used in combination with organic photodetectors to detect nitrite [68] or phosphate [70]. Alternatively, organic photovoltaic detectors have also been used [69]. Nevertheless, the limited radiation intensity and stability of OLEDs have so far limited their practical relevance for quantitative analytical tasks, which is also reflected in the few publications on OLEDs as light sources in photometric devices.

Finally, recent advances in microelectronics, sensor technology and data processing have significantly improved the capabilities of LED-based platforms. Historically, early photometers relied on photodiodes with transimpedance amplifiers, to convert light into a proportional voltage, which was then manually or computer-converted into absorbance units in accordance with the Beer–Lambert law. Today, programmable microcontrollers are commonly used for signal processing and system control in LED-based photometric devices. While logarithmic conversion for absorbance calculations is often implemented digitally, the achievable precision depends on the microcontroller architecture and numerical resolution. Consequently, high-accuracy measurements frequently rely on alternative approaches, such as analog logarithmic ratio amplifiers or direct current digitization, rather than purely software-based logarithmic conversion on low-resolution microcontrollers. Across the reported studies, successful implementation of LED-based photometric systems is rarely determined by the light source alone. Instead, analytical performance consistently depends on the combined optimization of wavelength selection, optical design, detector configuration, signal processing, and calibration strategy.

### 2.3. Detector Types, Configurations, and Signal Acquisition in LED-Based Analysis Systems

Detectors for analysis systems using LEDs as a light source must fulfill several requirements. Above all, the sensor must provide the spectral sensitivity at the specific wavelength of the light emitter LED, which is crucial for the desired signal retention and accuracy [12,14]. Furthermore, the detector should demonstrate a linear response with respect to light intensity over the relevant dynamic range, thereby ensuring accurate determination of the absorbance following logarithmic signal conversion according to the Beer–Lambert law. In addition, low noise, fast response time, long service life, low power consumption, compactness, low price and temperature stability are among the most important features [12,14,16,72]. Despite its fundamental importance, baseline noise is rarely reported consistently in the literature on LED-based photometric detectors, limiting objective comparison between different detector types and readout architectures.

Ambient-light interference can be reduced by appropriate optical shielding [73,74], while background contributions can be addressed by blank or reference measurements [75]. Appropriate analog front-end design and signal averaging can improve signal-to-noise performance, while LED intensity variations can be reduced by stable current control or reference measurements [76]. Sample-related effects, including inherent color, autofluorescence, and turbidity-induced scattering, may require wavelength-specific correction, matrix-specific calibration, or correction of scattering effects [75,77]. Fixed optical geometries and reproducible positioning of the LED, sample cell, and detector further minimize geometric variations. Thus, robust LED-based systems require coordinated optimization of optical design, signal acquisition, processing, and calibration.

Photodiodes are the most widely used detectors in LED-based analytical systems, particularly for measurements in the visible spectrum. Silicon photodiodes, in particular, offer sensitivity up to approximately 1000 nm and a fast response time, usually in the range of nanoseconds to microseconds. Their simple electrical design and robust performance make them ideal for both portable and high-precision instruments [11,72,78]. Specialized variants extend sensitivity into the UV or IR range [16]. Photodiodes were first used in an LED-based photometric setup by Anfält et al. (1976) to determine the alkalinity of seawater [3]. Since then, numerous devices have employed the LED–PD combination, with consistent success in various fields [11,15,55,58,78,79,80]. Other prominent sensor options include phototransistors (PTs), light-dependent resistors (LDRs), photomultiplier tubes (PMTs), and even LEDs themselves [12]. PTs offer higher sensitivity and internal signal amplification compared to PDs. However, their slower response times, typically in the microsecond to millisecond range and thermal drift, limit their suitability for applications requiring high-speed data acquisition or wide dynamic range [81,82]. They are preferred in low-light conditions where sensitivity is more critical than speed. LDRs are now rarely used in modern analytical instruments due to their slow response and lower sensitivity and are therefore not discussed in detail here [16]. Moreover, traditional LDRs often use cadmium sulfide, a toxic material, which has motivated the development of cadmium-free photoresistive materials, including organic semiconductor-based alternatives. PMTs are advantageous in systems where extremely low light intensities need to be detected with high precision, as they are highly sensitive light detectors and amplify the signal generated by the incident photons [83]. Fluorescence or bioluminescence measurements are examples for such low-light detection applications. However, due to the high operating voltage, fragility, size and high cost, they are less suitable for portable and low-cost use. Interestingly, LEDs can themselves be used as light detectors due to their ability to generate a photocurrent when exposed to light. Although their sensitivity is significantly lower and their spectral range narrower compared to photodiodes, this effect has been successfully exploited in paired emitter–detector diode (PEDD) systems [11,84,85]. First described in 2003 [85] and subsequently adapted several times in LED-based analytical systems [84,86,87,88], PEDDs are a minimalist and cost-efficient option for specific applications where simplicity is paramount. Complementary metal-oxide-semiconductor (CMOS) sensors are another type of detector used in LED-based optical systems, although they are less commonly employed, but merit mention. Table 1 provides an overview of the detectors used in LED-based analytical devices. Ultimately, the selection of the optimal sensor depends on the measurement principle, target wavelength, required sensitivity, and intended analytical task. Across the reviewed literature, photodiodes clearly emerge as the preferred detector technology owing to their favorable balance of sensitivity, stability, cost, and ease of integration. Alternative detector concepts, including PMTs and PEDDs, occupy more specialized niches where either maximum sensitivity or minimal system complexity is required.

## 3. Application Areas of LED-Based Photometers

The ongoing trend toward miniaturization in analytical chemistry has enabled the development of portable devices capable of performing analytical procedures directly on-site. In particular, several LED-based photometric instruments have demonstrated good agreement with conventional laboratory spectrophotometers for specific analytical parameters, including measured concentrations, calibration linearity, and detection limits, under the investigated experimental conditions [34,39,99]. As a result, these compact systems are increasingly being applied across a broad range of fields, including environmental monitoring, healthcare, food safety, education, process control and other (see Figure 3) [100,101,102,103,104,105]. For this overview, each reviewed publication was assigned to a single primary application field according to its main intended application. Although some technologies may be transferable to multiple fields, all reviewed studies could be unambiguously classified into one primary category. Rather than introducing new analytical chemistries, most reported systems combine established colorimetric or fluorimetric assays with compact, application-specific instrumentation. As a result, innovation in LED-based photometry is primarily driven by advances in instrument design, portability, automation, and system integration. This section reviews recent developments across these application areas to identify common design strategies, recurring analytical challenges, and emerging trends that extend beyond individual applications. By comparing different analytical fields, it becomes possible to identify application areas that are more advanced in terms of published research and those where further technological development and validation are still required.

### 3.1. Environmental Monitoring

Environmental monitoring is one of the most mature application areas for portable LED-based photometers, particularly for decentralized water-quality analysis. A wide range of assay methods has been developed for the determination of environmental pollutants using portable LED-based photometers. Target analytes include nutrients such as nitrate [106], nitrite [106,107], and phosphate [23,73], as well as heavy metals [22,25,35,108,109,110], herbicides [27,111], and total organic carbon (TOC) [112,113].

Most developed LED-based analysis systems for environmental monitoring are based on LEDs with emissions in the visible wavelength range for performing colorimetric assays [96,107,109,110,111,113], with RGB-LEDs occasionally being used to increase the flexibility of the devices [106,108]. Despite the increasing commercial availability of UV and NIR-LEDs, their analytical potential remains largely underexploited in environmental monitoring. Exceptions include, for example, the use of UV LEDs for the detection of TOC [76,112]. Absorbance measurements clearly dominate the field, whereas fluorescence-based implementations remain the exception despite their potential for improving analytical selectivity [76]. This methodological narrowness limits the chemical scope of LED-based devices and does not fully exploit the broader spectral possibilities now available due to advances in LED technology. Across the reviewed studies, environmental applications were predominantly evaluated using real water samples, although several methods relied on spiked samples for recovery studies or analytical validation. However, most systems were developed and validated for a single environmental matrix, such as river water [23,25,27,110], tap water [22,76,109], wastewater [106], rainwater [107] or seawater [114]. While this specialization allows analytical performance to be optimized for a specific sample matrix, it limits broader applicability and hinders use under heterogeneous or unpredictable field conditions. Systematic cross-matrix validation remained the exception. Notable examples include the RGB-LED system developed by Hoang et al. [106], which enabled multi-parameter evaluation within a single device, and the nitrite photometer reported by Khoshmaram et al. [107], which was successfully evaluated using multiple real-world water matrices, including surface water, tap water, snowmelt, and rainwater. Such studies illustrate the importance of broader matrix validation for demonstrating analytical robustness under realistic environmental conditions. Expanding the use of UV and NIR-LEDs, incorporating fluorescence modes, and validating across different water matrices could broaden the analytical scope of these systems.

Despite promising sensitivities, several shortcomings limit practical application. Interfering substances in complex environmental samples of different sample matrices are often neglected. Claims of “environmentally friendly” methods or working under the “Green Chemistry concept” [115] are sometimes undermined by the continued use of toxic reagents such as 1-naphthol [107], o-phenanthroline [114] or thiocyanate [25], raising questions about their suitability for field-use. Similarly, many methods developed with using an LED-based device require additional preparatory preconcentration steps such as a magnetic solid phase extraction [107], the use of a column packed of chelating resin [114] or nanoparticle reagents [35], which complicate workflows and limit feasibility for field deployment.

Another recurring observation across the reviewed studies is the discrepancy between the intended application and the actual validation strategy. Although many devices are described as field-deployable, true on-site validation remains uncommon. In most cases, environmental samples were collected in the field but analyzed later under controlled laboratory conditions. Only a few studies demonstrated measurements directly under real operating conditions, for example, those by Khoshmaram et al. [107], Seetasang et al. [111], and Hasegawa et al. [108]. Similarly, while instruments are frequently labeled as “portable,” detailed demonstrations of portability are rare, as information on dimensions or weight is often missing. Counterexamples include the studies of Koronkiewicz et al. [113] and Miranda et al. [25], which provide precise specifications to substantiate claims of portability. In this context, many authors emphasize the robustness and compactness of their devices without adequately considering actual field conditions or environmental influences on performance. Only a few studies systematically evaluate factors such as temperature effects on measurement accuracy [25], housing for electronic protection [35,114,116], or the stability of reagents under varying environmental conditions [111]. Analytical validation also varied considerably across the reviewed studies. While only few systems were validated against established reference methods, including such as HPLC [111], ICP-OES [109,114], or UV–Vis spectrophotometry [23], many others relied primarily on spike-and-recovery experiments to demonstrate analytical performance [27,106,107,108,111,113]. Although recovery studies provide valuable information on method accuracy, they cannot fully replace validation against independent reference methods under realistic sample conditions. Such comparisons are essential to demonstrate analytical reliability and to substantiate claims of applicability in real-world monitoring. Similar to claims of portability that are not supported by information on device size or weight, LED-based devices are often described as “low-cost” without providing concrete details on component expenses or overall production costs.

An overview of the most recently developed systems for environmental monitoring, including analytes of interest, applied assay methods, key system characteristics, and detection limits, is provided in Table 2. Overall, environmental monitoring represents one of the most extensively investigated application fields for LED-based photometers, with numerous systems demonstrating successful quantitative determination of specific analytes under the investigated conditions. However, broader cross-matrix evaluation and validation under real on-site conditions remain important requirements for demonstrating their robustness in environmental monitoring.

### 3.2. Health Monitoring

Point-of-care testing (POCT) has become one of the most important application areas for portable analytical devices, enabling diagnostic measurements directly at the site of patient care. Driven by the demand for rapid and decentralized diagnostics, LED-based photometers have increasingly been investigated as compact platforms for POCT applications. The global COVID-19 pandemic further accelerated interest in POCT, particularly due to its effectiveness in early pathogen detection and self-testing applications [117].

Representative systems illustrate both the promise and the current bottlenecks as POCT tools when combined with appropriate assay methods (see Table 3) [118]. Across the reviewed literature, innovation has been driven less by the development of new biochemical assays than by translating established diagnostic methods into portable, user-friendly analytical platforms suitable for decentralized healthcare. Although many of the proposed systems were evaluated using clinically relevant samples, including blood [119,120], serum [121,122,123], urine [94,124], saliva [125], or sweat [126], most remained at the proof-of-concept or early laboratory validation stage, whereas clinical pilot studies and routine clinical implementation were rarely reported. For pathogen detection, the 2-LED µ-spectrophotometer by Reuter et al. demonstrates the potential of the plasmonic gold nanoparticle assay for portable pathogen testing [74]. While the dual-channel design offers robustness against intensity fluctuations, the method remains at the proof-of-concept stage, with no field validation and limited consideration of nanoparticle stability. Similarly, colorimetric devices for hemoglobin estimation [119] or chloride in sweat for cystic fibrosis diagnosis [126] show use of RGB-LEDs and low-cost optics, but performance has so far been verified only on laboratory samples, with limited evidence on robustness against matrix interferences or clinical usability. Although some studies compared their results with commercial hospital analyzers [122], UV–Vis spectrometers [125], or routine clinical laboratory procedures [123,125], truly independent reference methods were applied only occasionally. However, these comparisons were usually performed against other photometric methods, whereas validation using independent analytical techniques or larger clinical studies remained uncommon. In the ultraviolet range, UV-LED devices for cortisol detection [127] or nitrite in urine [124] promise label-free quantification but face significant challenges: strong matrix effects in real fluids, lifetime issues, and performance saturation at higher analyte concentrations. In contrast, handheld spectrometers such as that by Maity et al. for malaria detection extend functionality through multivariate spectral classification [121], but the extremely high reported accuracies raise concerns of overfitting given the small datasets. More sophisticated systems, such as the handheld α-amylase colorimeter [125] or the pump-free LED–microfluidic platform for serum parameters [122], demonstrate progress toward integrated POCT workflows. However, their validation remains limited, and the complexity of liquid handling or reagent stability reduces their immediate applicability in low-resource or decentralized settings.

A major limitation across the health-monitoring studies is the limited transition from analytical proof-of-concept to clinical validation. Although clinically relevant samples were frequently investigated, larger clinical studies and systematic evaluation under realistic point-of-care conditions remained uncommon. Quantitative information supporting claims of portability was also inconsistently reported, although several studies provided device specifications or fabrication costs [74,94,119,122,126,128].

Overall, the reviewed studies demonstrate considerable progress towards translating established analytical methods into portable diagnostic platforms. Compared with the other application fields discussed in this review, health-monitoring studies more frequently included real clinical samples and comparisons with routine clinical laboratory instruments. However, the maturity of the reported systems varies considerably. Most devices remain at the laboratory prototype or early validation stage, with only limited evidence from clinical studies or routine application. Wider implementation will require rigorous clinical validation using representative sample populations and systematic evaluation under realistic point-of-care conditions.

### 3.3. Food Quality Monitoring

Monitoring food quality is essential for consumer safety and process control throughout the food industry. Because many quality parameters require rapid decision-making, LED-based photometric systems have increasingly been investigated for decentralized analysis of nutrients, contaminants, adulterants, and chemical residues.

**Table 2:** Examples of LED-based analytical system applications for environmental monitoring (LED = light emitting diode, PD = photodiode, PT = phototransistor, LDR = light-dependent resistor, PEDD = paired emitter–detector diode, PMT = photomultiplier tube, LOD = limit of detection, LR = linear range, n. s. = not specified).

Analyte	Sample	Sample-Handling Configuration	Assay or Method Used	λ_max_ of Employed LED	Sensor Type	Analytical Performance	Reference
Cd^2+^	River and tap water	MCFA	Reaction of Cd^2+^ with dithizone	LED: 533 nm	PT	LOD: 3.00 µg L^−1^ LR: 7.80–62.50 µg L^−1^ Recovery: 90–112%	[109]
Co^2+^	River and lake water	MCFA	Oxidization of Tiron by Co^2+^ and formation of a complex	LED: 425 nm	PD	LOD: 0.06 µg L^−1^ LR: 0.13–1.50 µg L^−1^ Recovery: 90–111%	[110]
Fe^2+^, Fe^3+^	River water	MCFA	Reaction of Fe^3+^ with thiocyanate	LED: 474 nm	PD	LOD: 0.08 mg L^−1^, 0.06 mg L^−1^ $\mathrm{LR}:\;0.10–2.00\;{mg}_{{Fe}^{2+}}L^{-1}$ Recovery: n. s.	[25]
Fe^3+^	Spiked samples of local tap water, sea water, well water	Batch	Solid phase extraction and o-phenanthroline method	Ultrabright white LED	CMOS	LOD: 5.00 µg L^−1^ LR: 10–1500 µg L^−1^ Recovery: 93–106%	[114]
Hg^2+^	River water, tap water and drinking water	MCFA and batch	Reaction of dithizone in a chloroform medium	LED: 485 nm	PD	LOD: 0.38 µg L^−1^ LR: 0.50–10 µg L^−1^ Recovery: 92–108%	[22]
NO_2_^−^	Tap water, snow and rainwater	Batch	Reaction with 4-nitroaniline and naphtha-1-ol	LED_1_: 625 nm, LED_2_: 525 nm, LED_3_: 465 nm	LDR	LOD: 1.30 µg L^−1^ LR: 0.01–0.30 mg L^−1^ Recovery: 87–103%	[107]
Pb^2+^	Spiked water samples	Batch	Gold nanoparticles (AUNPs)	White LED: 450–620 nm	PT	LOD: 890 µmol L^−1^ LR: 0.60–10 mmol L^−1^ Recovery: n. s.	[35]
Pq^2+^	Water sample of rice field	Batch	Reduction with sodium dithionite in alkaline medium	LED: 609 nm	PEDD	LOD: 0.50 mg L^−1^ LR: 2–40 mg L^−1^ Recovery: 89–105%	[111]
Pq^2+^	Spiked River water and tap water	MCFA	Reduction with sodium dithionite in alkaline medium	Red LED	LDR	LOD: 0.15 mg L^−1^ LR: 0.2–10 mg L^−1^ Recovery: 101–105%	[27]
PO_4_^3−^	Waste water, lake water, ground water	MCFA	Molybdenum blue reaction	LED: 630 nm	PEDD	LOD: 0.10 mg L^−1^ LR: 0.10–12 mg L^−1^ Recovery: 92–106%	[113]
PO_4_^3−^	Lake water	MCFA	Reaction with molybdate in acid medium	RGB-LED: 465 nm, 525 nm, 640 nm	PD	LOD: 0.02 mg_P_ L^−1^ LR: 0.15–3.10 mg_P_ L^−1^ Recovery: 96–111%	[23]
PO_4_^3−^	Lake water	Batch	Molybdenum blue reaction with commercially available test kit	590 nm	PT	LOD: 0.06 mg L^−1^ LR: 0.20–1 mg L^−1^ Recovery: n. s.	[73]
Tetracycline, N-NO_2_^−^, N-NO_3_^−^, Fe^2+^, Mn^2+^	Waste water samples	Batch	Commercial test kits	RGB-LED: 380–780 nm	PD	LOD: 0.88, 0.34, 0.03, 0.08, and 0.47 mg L^−1^ LR: 0–20, 0–17, 0–0.30, 0–1.75, 0–15 mg L^−1^ Recovery_tetracycline_: 92–101%	[106]
TOC	Water	Batch	Catalytic combustion of TOC to CO_2_	LED: 280 nm	PD + TIA	LOD: n. s. LR: n. s. Recovery: 95–99%	[112]
TOC	Tap water	Batch	Natural fluorescence of DOM	LED_1_: 280 nm, LED_2_: 335 nm	PMT	LOD: n. s. LR: <2.50 mg L^−1^ Recovery: n. s.	[76]
Turbidity	Turbidity standards	Batch	Attenuation of light due to turbidity	LED_1_: 900 nm, LED_2_: 850 nm	PEDD	LOD: 0.898 NTU LR: 0–100 NTU Recovery: n. s.	[129]
Trace As^3+^	Soil extract and hot-spring water samples	Batch	Molybdenum blue method with tetradecyldimethylbenzylammonium chloride on a cellulose ester membrane filter	RGB-LED: 470 nm, 530 nm, 630 nm	PD + PMT	LOD: 0.01 µg filter^−1^ LR: <0.50 µg filter^−1^ Recovery: 97%	[108]

Across the reviewed studies, most food-monitoring systems were evaluated using authentic food samples, including milk [130,131,132,133], wine [134,135], tea [135,136], spirits [137], fruit juices [134], and edible oils [138] obtained from local markets, farms, or production facilities. However, despite the use of realistic sample matrices, validation under routine industrial operating conditions remained uncommon. The reviewed studies demonstrate both the potential of these systems and their current limitations. For instance, portable devices for detecting ethanol in spirits [137] and urea in milk [132] illustrate how established analytical methods can be translated into compact food-monitoring systems. However, ethanol detection is currently limited to clear samples, whereas urea determination requires lengthy reaction times and costly reagents. Although heavy-metal assays such as those for copper in sugarcane spirits [139] and tin in canned foods [105] achieved impressive sensitivity, reagent instability and often demanding sample preparation reduce their practicality for decentralized food analysis. Other approaches demonstrate innovation in terms of portability and digital integration. NIR-photometers for detecting water adulteration in milk [133] produced robust results, but their reliance on expensive InGaAs sensors limits their affordability. Internet of Things (IoT)-enabled sensors for oxalate in tea [136] and PEDD-based chlorine detection in milk [131] are low-cost and connect to smartphones, but their long-term stability and selectivity under industrial conditions is unclear. High-throughput flow systems for polyphenols [135] and sugar content in cane juice [140] highlight the potential for process integration. However, the stability of reagents, e.g., hypochlorite, and the ruggedness of the systems under field conditions have not been systematically examined. Many studies compared their results with commercial spectrophotometers [131,132,136,139], ICP-OES [105], cryoscopic methods [133], or standardized analytical procedures [138,141]. Nevertheless, spike-and-recovery experiments remained the predominant validation strategy [131,136,138], whereas interlaboratory studies and routine industrial validation were rarely reported. Furthermore, a recurring limitation was the extensive sample preparation required prior to analysis. Depending on the analyte and food matrix, digestion [105], protein precipitation [132], extraction [138], or dilution steps were frequently necessary, reducing the practical advantages of otherwise portable instrumentation for rapid on-site analysis.

Overall, food quality monitoring has emerged as an important application area for LED-based photometry, with numerous studies demonstrating successful quantitative analysis of targeted analytes under the reported validation conditions. Across the reviewed literature, many systems successfully combine established colorimetric assays with compact instrumentation and often demonstrate good agreement with established laboratory methods for targeted analytes. However, practical implementation remains limited by extensive sample preparation, insufficient evaluation across different food matrices, and only limited validation under routine industrial conditions. Moreover, although matrix effects arising from fats, proteins, colorants, or turbidity were occasionally addressed through recovery experiments, systematic investigations across a broader range of food matrices remained uncommon. Quantitative reporting of device dimensions, weight, and fabrication costs also remained inconsistent, with only a few studies providing corresponding specifications [130,132,133,138,139]. Long-term reagent stability under routine food-processing conditions was also rarely evaluated. Finally, the use of toxic reagents in some approaches raises sustainability concerns. The use of hazardous reagents in some assays also raises practical and sustainability concerns for decentralized food analysis. An overview of the most recently developed systems for food monitoring is provided in Table 4.

**Table 3:** Examples of LED-based analytical system applications for health monitoring (LED = light emitting diode, PD = photodiode, PT = phototransistor, LDR = light-dependent resistor, PEDD = paired emitter–detector diode, PMT = photomultiplier tube, CMOS = complementary metal-oxide-semiconductor, LOD = limit of detection, LR = linear range, n. s. = not specified).

Analyte	Sample	Disease	Sample-Handling Configuration	Assay or Method Used	λ_max_ of Employed LED	Sensor Type	Analytical Performance	Reference
Cl^−^	Sweat	Cystic fibrosis	MCFA	Commercial colorimetric test kit	LED: 460 nm	PD	LOD: n. s. LR: 20–100 mmol L^−1^ Recovery: n. s.	[126]
Cortisol	Synthetic samples containing cortisol	Various diseases	Batch	Absorption of cortisol in UV region of the spectrum	LED1: 240 nm. LED_2_: 250 nm, LED_3_: 260 nm	PD	LOD: n. s. LR: n. s. Recovery: n. s.	[128]
Creatinine	Urine	Kidney disease, monitoring renal infections, chronic failure, drug effect	FIA	Jaffé reaction: with picric acid in alkaline medium	LED: 520 nm	PEDD	LOD: 0.90 mg L^−1^ LR: 100–500 mg L^−1^ Recovery: 91–118%	[94]
H1N1 virus	Synthetic samples containing virus	Swine flu	Plasmonic chip based on nanohole arrays	Extraordinary light transmission	White-color LED	CMOS	LOD: 10^3^ TCID_50_ m L^−1^ LR: n. s. Recovery: n. s.	[129]
Hemoglobin	Blood	Anemia	Batch	Cyanmethemoglobin method	RGB-LED set to 530 nm	PD	LOD: n. s. LR: n. s. Recovery: n. s.	[119]
Human pathogene (*Legionella pneumophila*)	n. s.	Legionellosis	Batch	Plasmonic nanoparticles and a DNA-amplification (LAMP) assay	LED_1_: 430 nm or 530 nm, LED_2_: 690 nm or 630 nm	PD	LOD: 0.50 pg µL^−1^ LR: n. s. Recovery: n. s.	[74]
Malaria parasite identification factors	Human blood serum	Malaria	Batch	Parasite identification factors using molecule level optical scattering information	White LED	CMOS	LOD: n. s. LR: n. s. Recovery: n. s.	[121]
NO_2_^−^	Simulated urine	Urinary tract infection	Batch	Maximum absorption characteristics of nitrite at 354 nm	LED: 370 nm	PD	LOD: n. s. LR: n. s. Recovery: n. s.	[124]
PO_4_^3−^, Ca^2+^	Human serum	Hyperphosphatemia	Paper-based	Phosphate: reaction with ammonium molybdate, calcium: formation of a complex with EDTA	LED_PO4_^3−^: 525 nm, LED_Ca2+:_ 470 nm	LED_PO4_^3−^: 655 nm, LED_Ca2+:_ 630 nm	LOD: 1.40 µmol L^−1^, 7.40 µmol L^−1^, LR: 1–70 µmol L^−1^, 10–100 µmol L^−1^ Recovery: 100–103%, 99–100%	[123]
TnI (troponin subunit)	Whole blood and plasma	Stroke and other myocardial injuries	Batch	Antibodies marked with europium chelate label	LED: 340 nm	PD + PMT (fluorescence detected at 615 nm)	LOD: n. s. LR: n. s. Recovery: n. s.	[120]
Triglyceride (TGL), albumin (ALB) and total protein (TP)	Human blood serum	Diseases of liver, kidney, or bone marrow, metabolic disorders (ALB and TP), atherosclerosis, hypertension, liver disease and more (TGL)	Microfluidic chip	Various	LED_ALB_: 600 nm, LED_TGL_: 510 nm, LED_TP_: 540 nm	PD	LOD: 0.10 mmol L^−1^, 4 g L^−1^, 10 g L^−1^ LR: 0.1–5.45 mmol L^−1^, 25–60 g L^−1^, 50–95 g L^−1^ Recovery: n. s.	[122]
α-amylase activity	Saliva	Stress assessment and stress-related diseases	Batch	3,5 dinitrosalicylic acid (DNS) assay	LED: 525 nm	n. s.	LOD: 0.10 U mL^−1^ LR: 0.10–1.000 U mL^−1^ Recovery: n. s.	[125]

**Table 4:** Examples of LED-based analytical system applications for food monitoring (LED = light emitting diode, PD = photodiode, PT = phototransistor, LDR = light-dependent resistor, PEDD = paired emitter–detector diode, PMT = photomultiplier tube, LOD = limit of detection, LR = linear range, n. s. = not specified).

Analyte	Sample	Sample-Handling Configuration	Assay or Method Used	λ_max_ of Employed LED	Sensor Type	Analytical Performance	Reference
Acidity	Fruit juice, vinegar and wine	MCFA	Titration with phenolphthalein as indicator dye	LED: 530 nm	PD	LOD: n. s. LR: 0.036–0.176 mg L^−1^ (titration) Recovery: n. s.	[134]
Cu^2+^	Sugarcane spirits	Batch	Chelation reaction between Cu^2+^ and cuprizone	LED: 597 nm	PD	LOD: 0.200 mg L^−1^ LR: 1–12 mg L^−1^ Recovery: 97–104%	[139]
Ethanol	Distilled ethanolic beverages	MCFA	Schlieren effect	LED_1_: 470 nm, LED_2_: 525 nm, LED_3_: 627 nm	PD	LOD: 0.80% v v^−1^ LR: 2.50–60% v v^−1^ Recovery: n. s.	[137]
Fe^2+^	Vitamin tablets	Batch	o-Phenanthroline method	LED: 464 nm	PT	LOD: 0.06 mg L^−1^ LR: 0.20–10 mg L^−1^ Recovery: n. s.	[141]
Deterioration of bleachability index (DOBI) and carotenoid content	Red palm oil	Batch	Absorption of light by specific compounds	LED_1_: 269 nm, LED_2_: 446 nm	PD	LOD: n. s. LR: n. s. Recovery: n. s.	[138]
Fat content	Raw natural milk	Batch	Light scattering by milk fat globules	RGB-LED: 466 nm, 531 nm, 631 nm	PD	LOD: n. s. LR: 1.5–3.2% Recovery: n. s.	[130]
Oxalate	Tea infusions	Batch	Reaction between oxalate and the red-colored Zr^4+^–SPADNS complex	LED: 590 nm	PD	LOD: 0.20 mg L^−1^ LR: 0.30–10 mg L^−1^ Recovery: 97–110%	[136]
Polyphenol (gallic acid)	Wine and tea	MCFA	Folin–Ciocalteu’s reagent method	LED: 490 nm	PD	LOD: 21 mg L^−1^ LR: 62–1000 mg L^−1^ Recovery: 96–102%	[135]
Sn^4+^	Canned food	MCFA	Reaction of Sn^4+^ with pyrocatechol violet (PCV) in the presence of surfactants	LED: 630 nm	PD	LOD.: 0.04 mg L^−1^ LR: 0.10–1.25 mg L^−1^ Recovery: 95–110%	[105]
Sodium hypochlorite for milk adulteration (expressed as Cl_2_)	Milk	Batch	Reaction with N, N-diethyl-p-phenylenediamine sulfate (DPD) aqueous solution of pH 6	White LED	PEDD	$\mathrm{LOD}:\;0.14\;{mg}_{{Cl}_{2}}L^{-1}$ $\mathrm{LR}:\;0.50–20\;{mg}_{{Cl}_{2}}L^{-1}$ Recovery: 107–114%	[131]
Sugar	Sugarcane plants	Batch	Direct absorption	LED_1_: 620 nm, LED_2_: 545 nm, LED_3_: 460 nm	PD	LOD: n. s. LR: n. s. Recovery: n. s.	[140]
Urea	Milk	Batch	Berthelot’s reaction	LED: 639 nm	PD	LOD: 0.18 mmol L^−1^ LR: 1–30 mmol L^−1^ Recovery: 96–105%	[132]
Water content for milk adulteration	Milk	MCFA	Light scattering effects with different water contents/ direct absorption	LED_1_: 970 nm, LED_2_: 1450 nm, LED_3_: 1200 nm	PD	LOD: n. s. LR: n. s. Recovery: n. s.	[133]

### 3.4. Industrial Applications and Product Quality Monitoring

Beyond food quality monitoring, industrial process monitoring represents another important application area for LED-based photometers. In biotechnology, pharmaceutical manufacturing, and the chemical industry, rapid and decentralized measurements support process optimization, quality assurance, and real-time decision-making. Due to their compact design and affordability, LED-based systems facilitate the adoption of process analytical technologies, particularly in small and medium-sized enterprises. Compared to the other application areas examined, the focus in industrial applications is less on portability and more on automation and flow-based analysis with a view to potential process integration. Across the reviewed studies, most systems were evaluated using industrially relevant samples or products rather than during continuous operation in real production environments.

LED-based MCFA systems have been successfully adapted for pharmaceutical quality assurance, such as the determination of losartan potassium in tablets [142]. The developed procedure combined automated analysis with a broad linear range and showed no significant difference from the HPLC reference method for the analyzed pharmaceutical samples. However, the study was conducted under laboratory conditions, and robustness under industrial operating conditions has yet to be demonstrated. NIR-LEDs have been used for ethanol quantification in gasoline and for detecting adulteration in hydrated ethanol fuel [75,143]. These approaches exploit the absorption bands of the overtones of the OH groups and, in some cases, combine low-cost LEDs with consumer-grade detectors, such as webcams. While these methods are attractive for low-cost screening, the sensitivity and selectivity are limited, and validation often relies on model mixtures rather than real-world samples from fuel stations. Automated titration-based LED photometers for measuring ethanol acidity [144] generate less chemical waste than standard titration methods. However, the complexity of the required pumps and valves reduces portability. In biotechnology, LED photometers are commonly used to monitor microbial growth via turbidity. Lucidi et al. [77] demonstrated that correcting for scattering effects using a geometrical optics model improves the accuracy of LED-based optical density readings, resulting in good agreement with commercial benchtop spectrophotometers and microplate readers over an OD_600_ range of 0.1–1.5. However, while such correction approaches can improve agreement with reference measurements, turbidity-based monitoring remains limited in terms of specificity, and validation has largely been restricted to controlled laboratory cultures rather than complex industrial matrices. In addition to liquids, mid-infrared (MIR)-LEDs have been tested for gas-phase analysis, including the simultaneous detection of CO and CO_2_ in engine exhausts [145]. These systems demonstrate the potential of LEDs as a low-cost alternative to lasers for real-time exhaust monitoring. However, there are high optical losses and limited sensitivity for CO, and no vehicle-based validation has yet been performed. Compared with environmental and food-monitoring studies, industrial applications more frequently emphasized automation, high analytical throughput, and reduced reagent consumption rather than field portability. Several flow-based systems explicitly addressed Green Chemistry aspects by minimizing reagent consumption and waste generation. However, these benefits were frequently offset by extensive sample preparation or laboratory-based validation, limiting their applicability for routine industrial monitoring.

Several additional limitations can be identified across the discussed industrial applications. Although many prototypes demonstrated successful quantitative measurements under the investigated laboratory conditions, their robustness under realistic process conditions, including fluctuating temperatures, complex sample matrices, and continuous operation, remains largely unexplored. Furthermore, calibration strategies were only rarely discussed in detail. Most studies relied on conventional calibration procedures, whereas aspects such as long-term drift compensation, flow-cell fouling, or adaptive calibration under changing process conditions were largely not addressed. For mid-infrared applications, the performance of LEDs is weak and there are optical losses, which further limit sensitivity compared to laser-based PAT tools. Furthermore, in regulated areas such as the pharmaceutical industry, few systems meet the stringent validation requirements of FDA guidelines, posing a significant hurdle to implementation. Overall, the reviewed studies illustrate the feasibility of applying LED-based photometers to industrially relevant analytical problems. Although agreement with established laboratory reference methods, including HPLC [142], IC and ICP-OES [146], FAAS [147], or commercial spectrophotometers [77], has been demonstrated in several studies, most systems remain validated only under laboratory conditions. Their suitability for continuous operation under routine industrial conditions therefore remains to be demonstrated. Addressing these requirements will be essential for establishing LED-based photometers as reliable tools within modern process analytical technologies and Industry 4.0 environments. Table 5 provides an overview of LED-based analytical systems for industrial applications and product quality monitoring.

### 3.5. Educational Purposes

LED-based photometers have become increasingly popular in STEM (science, technology, engineering, and mathematics) education because they combine low-cost instrumentation with transparent system architecture. Unlike commercial instruments, their open design enables students to directly explore the interaction between light sources, optical components, detectors, and signal processing, thereby transforming otherwise complex analytical instruments into accessible teaching tools [104,148,149]. This transparency promotes hands-on, inquiry-based learning and supports a deeper understanding of core concepts in optics and analytical chemistry. Students can explore light absorption, transmission, reflection, and the Beer–Lambert law in a practical context [149]. Additionally, working with different LEDs helps students understand how specific wavelengths influence color and absorbance, deepening their grasp of the electromagnetic spectrum [150]. Studies have shown that such hands-on, inquiry-based learning approaches positively affect students’ scientific literacy and self-regulation skills [151,152,153]. It should be noted that a cuvette is often used to measure samples in photometers for educational purposes in order to keep the complexity of the devices low. Only a few articles were found in which a photometer with a flow cell was described [154,155]. Across the reviewed studies, educational value arose primarily from transparent instrument design rather than maximum analytical performance. Many systems deliberately employed open constructions based on breadboards [156], Lego bricks [157,158], cardboard housings [159], or simple electronic components [154,160,161], enabling students to understand optical alignment, detector principles, signal processing, and calibration through hands-on experimentation.

Wang et al. [162] guided students in programming their own submersible photometer for acid–base titrations. More unconventional approaches include smartphone-integrated systems [159,163], which extend learning into digital and mobile platforms. These implementations span practical applications such as water and food analysis, pigment characterization, and even forensic dye detection, all of which highlight real-world relevance and sustain student engagement. Although nearly all studies reported educational benefits, only Chen et al. formally evaluated learning outcomes using educational assessment methods such as pre- and post-tests [160]. In most cases, successful classroom implementation was used as indirect evidence of educational effectiveness rather than systematically measuring learning gains. Several educational concepts deliberately incorporated authentic everyday samples, including natural waters [156], food products [157,158], pharmaceuticals [158], and cosmetic products [164], thereby strengthening the connection between analytical chemistry and real-world applications.

Limits of detection and analytical accuracy are often not emphasized, which could limit the devices’ applicability in more advanced or professional contexts. Accordingly, analytical validation was usually limited to comparison with commercial UV–Vis spectrophotometers rather than comprehensive validation studies, reflecting that educational systems primarily aim to illustrate analytical principles instead of maximizing analytical performance. For example, many educational LED-based photometers rely on inexpensive detectors, such as photoresistors, which introduce nonlinearity and signal drift. Device housings were often constructed from inexpensive materials [155,164,165], which lowered the entry barrier but also reduced robustness and reproducibility. Another concern is safety: some projects use hazardous reagents such as hydrazine [166] or UV LEDs [158] without adequate shielding, which raises questions about their suitability for teaching environments. Additionally, the variability in design quality and the lack of standardized methodologies can pose reproducibility challenges across different educational settings. Overall, educational LED-based photometers occupy a complementary role to laboratory spectrometers by providing affordable and transparent platforms for teaching analytical chemistry, spectroscopy, and instrument design. Their value therefore lies primarily in promoting experimental understanding and engineering skills rather than maximizing analytical performance. Future efforts should focus on reproducible open-source designs and curricula, appropriate safety concepts, and more systematic assessment of learning outcomes. Examples of LED-based analytical systems developed for educational purposes are summarized in Table 6.

## 4. Conclusions

LED-based photometers have evolved into versatile analytical platforms for decentralized chemical analysis across environmental monitoring, healthcare, food analysis, industrial process monitoring, and education. Across the reviewed studies, a consistent observation is that successful LED-based photometers are not defined by the use of LEDs alone, but by the careful integration of appropriate wavelength selection, optical design, detector technology, calibration strategies, and application-specific optimization. Recent developments were driven less by the introduction of entirely new analytical assays than by the successful translation of established photometric methods into compact, application-oriented devices. Despite these advances, several recurring challenges remain across the reviewed application fields. Reliable analytical performance depends on robust calibration, reproducible optical alignment, and adequate consideration of matrix effects under realistic operating conditions. Limited cross-matrix validation, inconsistent reporting of key performance parameters, and insufficient standardization continue to hamper objective comparison between different photometric concepts. Based on these recurring gaps, Table 7 summarizes recommended minimum reporting information for future studies to facilitate comparison, reproducibility, and assessment of practical applicability.

Only a minority of studies validated their systems against independent analytical reference methods such as HPLC, IC, ICP-OES, or FAAS, whereas many relied on comparisons with commercial photometric instruments or spike-and-recovery experiments. Systematic validation under realistic operating conditions also remained uncommon. The relevance of these challenges, however, differs between application fields. Environmental monitoring particularly requires robustness across variable natural matrices and field conditions, while healthcare applications depend on rigorous clinical validation using representative sample populations. Food and industrial applications require broader evaluation under routine production or process conditions, whereas educational applications place greater emphasis on accessibility, reproducibility, safety, and didactic value. Future developments should therefore combine technological advances, including multispectral detection, microfluidic integration, IoT connectivity, AI-assisted data analysis, and autonomous monitoring, with improved reproducibility, transparent reporting of analytical performance and relevant hardware implementation details, standardized validation, and reliable long-term operation. The establishment of common evaluation guidelines together with open-source hardware and software resources could further improve comparability and facilitate wider adoption. Overall, progress toward reliable decentralized analysis will depend on combining technological innovation with robust, application-specific validation under realistic operating conditions.

## Figures and Tables

**Figure 1:**
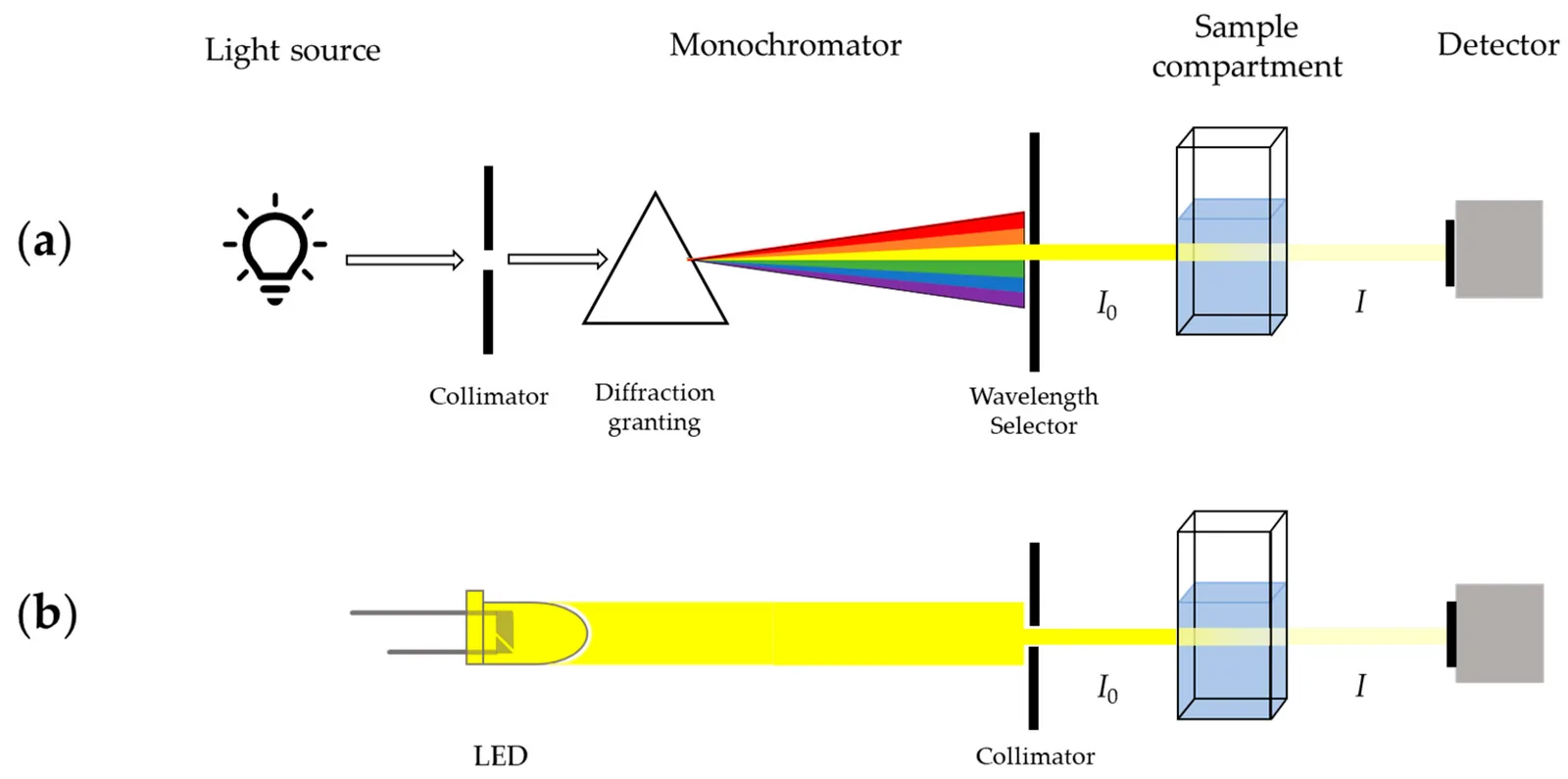
(**a**) Absorption spectroscopy with a 180° light path measuring intensity before (I_0_) and after (I) sample. (**b**) LED-based photometer setup without monochromator due to fixed emission wavelength of LEDs. The arrows indicate the direction of the light beam. The different colors represent different wavelengths, with the yellow beam representing the wavelength selected for the measurement.

**Figure 2:**
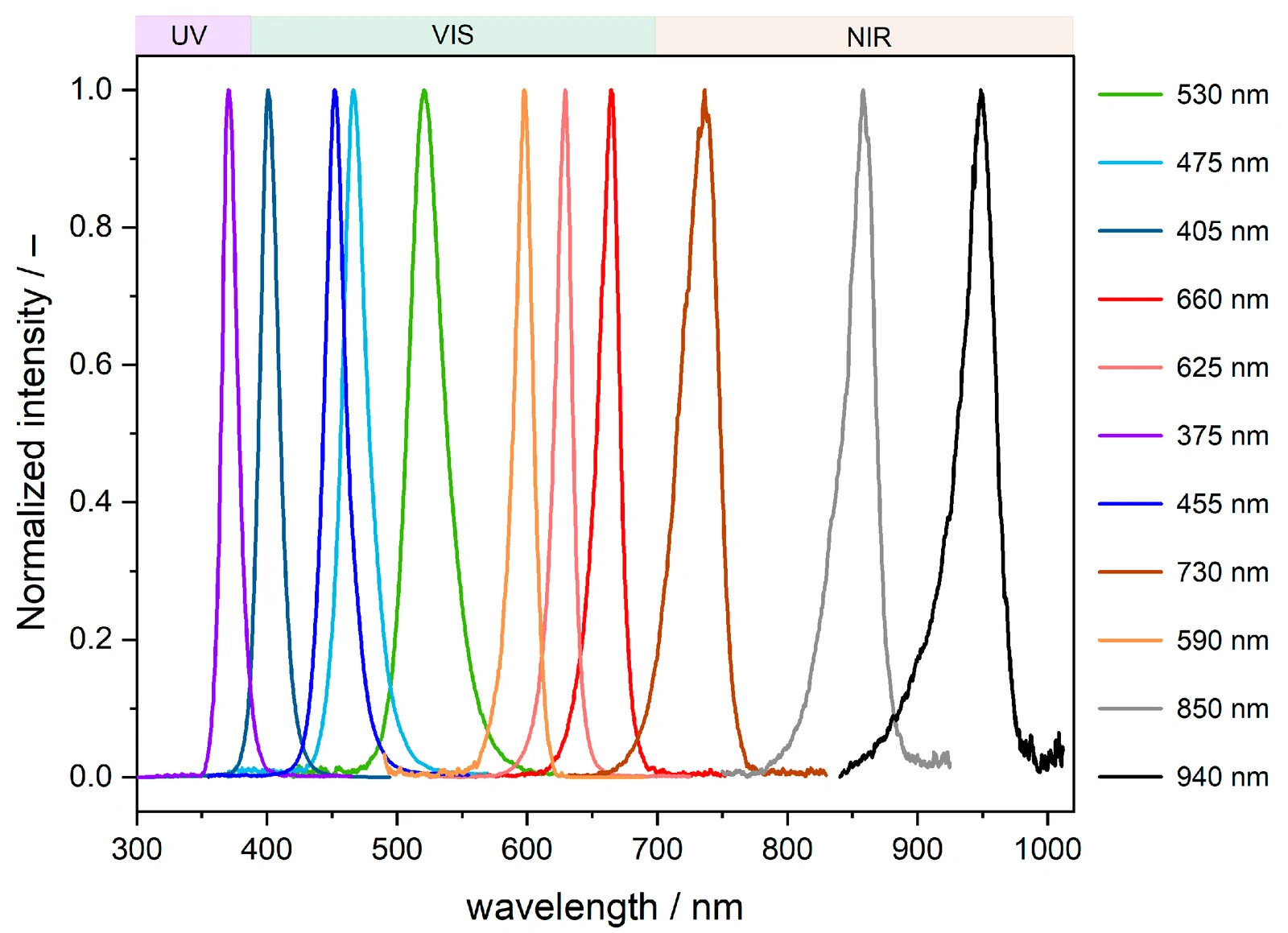
Examples of commercially available LEDs in the UV–Vis–NIR-spectrum range [47].

**Figure 3:**
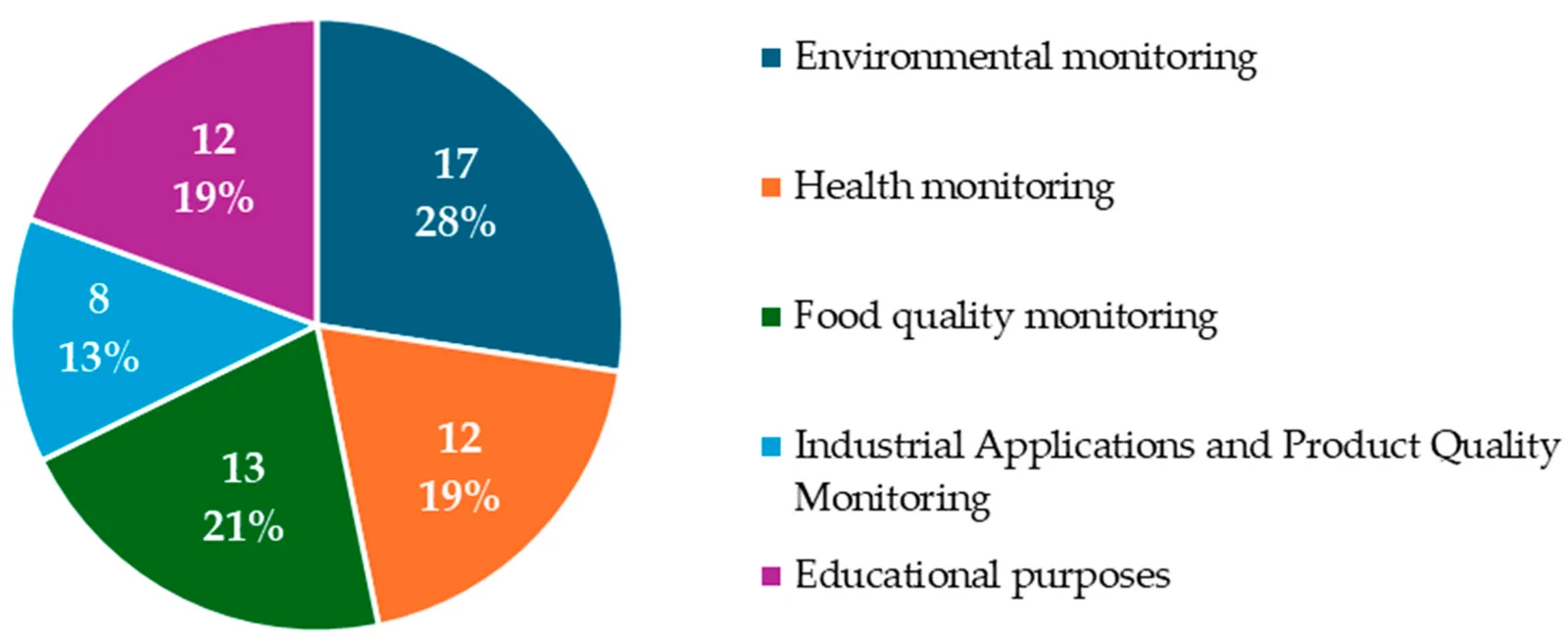
Distribution of publications included in the application-oriented comparative analysis presented in Section 3 across the five application fields (2015–2025; *n* = 62). Absolute numbers and relative proportions are reported for each category. Each publication was assigned to a single primary application field based on its main intended application and was counted only once. The distribution reflects only the publications included in this review and should not be interpreted as representing the entire literature within these application fields.

**Table 1:** Overview of the common detectors used in LED-based analytical devices. (* schematic representation of one pixel in an array).

Detector Type	Photodiode (PD)	Phototransistor (PT)	Light Emitting Diode (LED)	Photomultiplier Tube (PMT)	CMOS Image Sensors
Image and circuit diagram	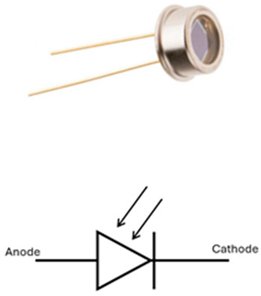	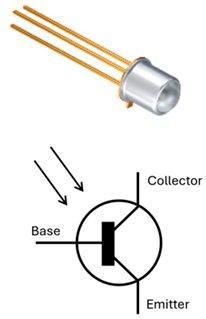	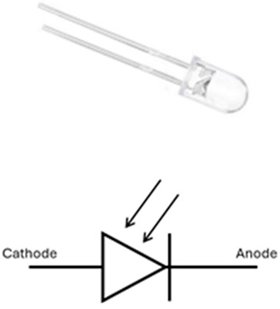	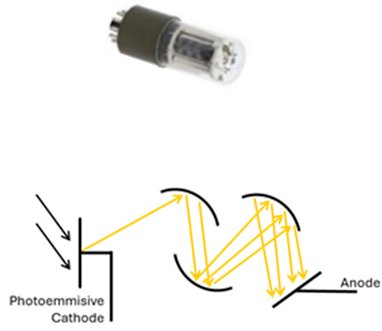	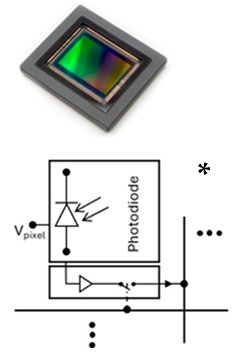
Measurement output	Depends on: PD + integrated or external transimpedance amplifier (TIA); PD + log-ratio amplifier (LRA); PD + direct current digitization.	Collector current: µA–mA; Often converted into a voltage across a resistor; Current → Voltage.	Time-to-discharge; Typically, less efficient and inferior sensitivity compared to photodiodes.	Anode current (pA–nA), converted to voltage via external transimpedance amplification; Requires external low-noise current-to-voltage conversion.	Digital intensity values from a pixel array of photodiodes; Multiple wavelength channels when combined with optical filters or dispersion elements.
Key characteristics	Reduced noise at low photocurrents; Improved thermal and electrical stability; LRA: logarithmic ratio output; TIA: output voltage ∝ light intensity within the linear range; Direct current digitization: conversion of photocurrent into digital signal.	Non-linear, but higher sensitivity than PDs; Thermal drift; Light at base increases current form collector; Signal is amplified internally.	Limited dynamic range and narrow detection bandwidth; Requires precise alignment and matching with the emitter wavelength; Used to avoid ADCs in low-cost systems.	High sensitivity, fast, suitable for detecting very low light levels; Electrons are multiplied across dynodes (10^6^–10^7^ gain); Requires high-voltage power supply (often 500–1500 V).	Linear within the pixel’s full-well capacity; saturation occurs at high light levels; Response time is typically in the µs to ms range per pixel; Signal readout via on-chip charge integration and amplification.
Typical areas of application	High-precision photometers with linear, fast response [89,90]	Medium-precision, cost-sensitive applications, e.g., educational or demonstrative [90,91,92]	DIY-projects, dual emitter–detector configurations [93,94]	Ultra-low light detection, e.g., fluorescence, bioluminescence, scientific research [95,96]	Multi-wavelength or spatially resolved measurements, imaging spectroscopy [97,98]

**Table 5:** Examples of LED-based analytical systems for industrial applications and product quality monitoring (LED = light emitting diode, PD = photodiode, PT = phototransistor, LDR = light-dependent resistor, PEDD = paired emitter–detector diode, PMT = photomultiplier tube, CMOS = complementary metal-oxide-semiconductor, LOD = limit of detection, LR = linear range, n. s. = not specified).

Analyte	Sample	Sample-Handling Configuration	Assay or Method Used	λ_max_ of Employed LED	Sensor Type	Analytical Performance	Reference
Acidity	Fuel ethanol	MCFA	α-naphtholphthalein as indicator	LED_1_: 647 nm; LED_2_: 530 nm	PD	LOD: n. s. LR: n. s. Recovery: 94–107%	[144]
Ag^+^	Sulphadiazine cream (radiology effluent)	Batch	Sensor prepared by immobilization of 5-(p-dimethylaminobenzylidene)-rhodanine (PDR) on triacetyl cellulose (TAC) membrane	Double beam system, LED: 560 nm	LDR	LOD: 0.20 µg mL^−1^ LR: 0.50–6 µg mL^−1^ Recovery: n. s.	[147]
CO, CO_2_	Engine exhaust gases	MCFA	Direct absorbance	LED_1_: 3.6 µm, LED_2:_ 4.2 µm, LED_3_: 4.7 µm	Photovoltaic PD	LOD: 400 ppm, 30 ppm LR: n. s. Recovery: n. s.	[145]
Ethanol	Gasoline	Batch	Direct absorbance	LED_1_: 970 nm, LED_2_: 1450 nm, LED_3_: 1530 nm	PD	LOD: 5.07% (v v^−1^) LR: n. s. Recovery: n. s.	[75]
Losartan potassium (Los-K)	Pharmaceuticals	MCFA	Light scattering (turbidimetric) particles formed by reaction losartan potassium with copper ions	LED: 385 nm	PD	LOD: 12.08 mg L^−1^ LR: 23.20–417.60 mg L^−1^ Recovery: n. s.	[142]
OD of bacterial cultures	Bacterial cultures	Batch	Geometrical optics approach	LED: 600 nm	CMOS	LOD: OD: 1.5 LR: n. s. Recovery: n. s.	[77]
SO_4_^2−^, Cl^−^	Petroleum coke	MCFA	Turbidimetric method for sulfate determination and mercury/thiocyanate method for chloride determination	LED: 472 nm	PD	LOD: 5.30 mg L^−1^, 0.16 mg L^−1^ LR: 10–700 mg L^−1^, 0.25–10 mg L^−1^ Recovery: n. s.	[146]
Water or methanol (as adulteration)	Hydrated ethyl alcohol fuel (HEAF)	Batch	Direct absorption	LED_1_: 805 nm, LED_2_: 845 nm, LED_3_: 902 nm, LED_4_: 912 nm, LED_5_: 930 nm, LED_6_: 1173 nm, LED_7_: 1282 nm	CMOS	LOD: 1% v v^−1^ LR: n. s. Recovery: n. s.	[143]

**Table 6:** Examples of LED-based analytical systems for educational purposes (LED = light emitting diode, PD = photodiode, PT = phototransistor, LDR = light-dependent resistor, PEDD = paired emitter–detector diode, PMT = photomultiplier tube, LOD = limit of detection, LR = linear range, n. s. = not specified).

Analyte	Sample	Sample-Handling Configuration	Assay or Method Used	λ_max_ of Employed LED	Sensor Type	Analytical Performance	Reference
Fe^2+^	Surface water samples	Batch	Complexation with iron indicator reagent 2,4,6-tripyridyl-s-triazine (TPTZ)	LED: 585 nm	LED	LOD: 0.022 mg L^−1^ LR: n. s. Recovery: n. s.	[156]
Folic acid; cinnamaldehyde	Folic acid tablets; cinnamon	Batch	Direct absorbance	LED: 355 nm	LED	LOD: n. s. LR.: n. s. Recovery: n. s.	[158]
Glucose	Test samples	Batch	Enzymatic reaction detection with glucose oxidase	LED: 425 nm	PT	LOD: n. s. LR: n. s. Recovery: n. s.	[160]
K_a_ (Equilibrium constant) of acid–base system	Synthetic buffer samples	Batch	Color change with bromothymol blue	RGB-LED: 463 nm, 525 nm, 645 nm	PD + CMOS	LOD: n. s. LR: n. s. Recovery: n. s.	[163]
Ni^2+^; Co^2+^	Test/synthetic samples	Batch	Commercial reagents	RGB-LED	Smartphone detector (Image J analysis)	LOD: n. s. LR: n. s. Recovery: n. s.	[159]
pH	Acidic/basic solution	Flow cell	Photometric titration with phenolphthalein, phenol red, bromocresol green, and bromothymol blue	Yellow LED, Green LED	PD	LOD: n. s. LR: n. s. Recovery: n. s.	[154]
PO_4_^3−^; food dyes; salicylate	Mixture of tartrazine and amaranth; water, soil and manure; acne face wash	Batch	Direct absorption; molybdenum blue method; complexation with Fe(III)	LED_1_: 470 nm; LED_2_: 524 nm; LED_3_: 640 nm	PD	LOD: n. s. LR: n. s. Recovery: n. s.	[164]
Quinine	Urine after drinking tonic water	Batch	Fluorescence	LED: 355 nm	LED	LOD: n. s. LR: <10 mg L^−1^ Recovery: n. s.	[157]
Red blood cell concentration; hemoglobin	Test/synthetic samples	Flow cell	Enzyme reaction with beta-galactosidase	LED_1_: 395 nm, LED_2_: 525 nm, LED_3_: 620 nm	LDR	LOD: n. s. LR: n. s. Recovery: n. s.	[155]
Salicylate	Commercial face wash	Batch	Complexation with Fe^3+^	LED: 535 nm	LDR	LOD: n. s. LR: 100–500 mg L^−1^ Recovery: n. s.	[161]
Turbidity	Yeast respiration experiment, kaolinite and montmorillonite clay suspensions	Batch	Reaction of Ca(OH)_2_ with CO_2_ from yeast respiration, flocculation (coagulation/sedimentation) of clay particles	IR-LED: 860 nm	IR-LED or LDR	LOD: n. s. LR: n. s. Recovery: n. s.	[167]
Turbidity	Synthetic samples	Batch	Reaction of formazine with hydrazine sulfate	LED: 470 nm	LDR	LOD: 2.7 NTU LR: n. s. Recovery: n. s.	[166]

**Table 7:** Recommended minimum reporting information for future studies on LED-based photometric systems.

Category	Recommended Minimum Information
Analytical performance	Linear range, LOD/LOQ, precision, recovery/accuracy
Calibration	Calibration model, number/range of standards, recalibration procedure
Optical system	LED wavelength/FWHM, detector type, optical geometry
Stability	Short- and/or long-term signal stability and drift
Thermal management	Operating temperature, thermal stabilization, and temperature compensation, if applicable
Device specifications	Dimensions, weight, portability, power requirements, housing/materials, optical alignment and light-shielding strategy
Cost	Approximate component or fabrication cost
Sample validation	Sample type and number; real, synthetic, or spiked samples
Reference validation	Reference method and agreement/statistical comparison
Practical validation	Field, clinical, or industrial testing status beyond proof-of-concept evaluation

## Data Availability

No new data was created or analyzed in this study. Data sharing is not applicable to this article.

## Abbreviations

The following abbreviations are used in this manuscript:

ADC	Analog–digital converter
AlGaN	Aluminum gallium nitride
AUNP	Gold nanoparticle
BTEX	Mixture of benzene, toluene, ethylbenzene and xylene
CMOS	Complementary metal-oxide-semiconductor
DIY	Do-it-yourself
FIA	Flow-injection analysis
FWHM	Full width at half-maximum
GaAs	Gallium arsenide
GaP	Gallium phosphide
HPLC	High-Performance Liquid Chromatography
ICP-OES	Inductively Coupled Plasma Optical Emission Spectroscopy
InGaN	Indium gallium nitride
IR	Infrared
IoT	Internet of Things
LED	Light emitting diode
LDR	Light-dependent resistor
MCFA	Multicommuted flow analysis
NIR	Near-infrared
OLEDs	Organic light emitting diodes
PAT	Process analytical technology
PD	Photodiode
PEDD	Paired emitter–detector diode
PMT	Photomultiplier tube
POCT	Point-of-care testing
PT	Phototransistor
RGB	Red–green–blue
STEM	Science, technology, engineering, and mathematics
TOC	Total organic carbon
UV	Ultraviolet
Vis	Visible
